# LPS binding protein and activation signatures are upregulated during asthma exacerbations in children

**DOI:** 10.1186/s12931-023-02478-3

**Published:** 2023-07-12

**Authors:** Anya C. Jones, Jonatan Leffler, Ingrid A. Laing, Joelene Bizzintino, Siew-Kim Khoo, Peter N. LeSouef, Peter D. Sly, Patrick G. Holt, Deborah H. Strickland, Anthony Bosco

**Affiliations:** 1 Wal-yan Respiratory Research Centre, Telethon Kids Institute, University of Western Australia, Perth, WA Australia; 2 UWA Medical School, University of Western Australia, Nedlands, WA Australia; 3 Division of Cardiovascular and Respiratory Sciences, The University of Western Australia, Perth, WA Australia; 4 Child Health Research Centre, The University of Queensland, Brisbane, QLD Australia; 5 Asthma & Airway Disease Research Center, The BIO5 Institute, The University of Arizona, Rm. 329, 1657 E. Helen Street, Tucson, AZ 85721 USA; 6 Department of Immunobiology, The University of Arizona College of Medicine, Tucson, AZ USA

**Keywords:** Atopic asthma, Network analysis, Bulk RNA-Seq, Peripheral blood, LPS, TGFB1

## Abstract

Asthma exacerbations in children are associated with respiratory viral infection and atopy, resulting in systemic immune activation and infiltration of immune cells into the airways. The gene networks driving the immune activation and subsequent migration of immune cells into the airways remains incompletely understood. Cellular and molecular profiling of PBMC was employed on paired samples obtained from atopic asthmatic children (n = 19) during acute virus-associated exacerbations and later during convalescence. Systems level analyses were employed to identify coexpression networks and infer the drivers of these networks, and validation was subsequently obtained via independent samples from asthmatic children. During exacerbations, PBMC exhibited significant changes in immune cell abundance and upregulation of complex interlinked networks of coexpressed genes. These were associated with priming of innate immunity, inflammatory and remodelling functions. We identified activation signatures downstream of bacterial LPS, glucocorticoids and TGFB1. We also confirmed that LPS binding protein was upregulated at the protein-level in plasma. Multiple gene networks known to be involved positively or negatively in asthma pathogenesis, are upregulated in circulating PBMC during acute exacerbations, supporting the hypothesis that systemic pre-programming of potentially pathogenic as well as protective functions of circulating immune cells preceeds migration into the airways. Enhanced sensitivity to LPS is likely to modulate the severity of acute asthma exacerbations through exposure to environmental LPS.

**Supplementary Information:**

The online version contains supplementary material available at 10.1186/s12931-023-02478-3.

## Introduction

Exacerbations of asthma in children are most commonly caused by acute respiratory viral infections. The immune mediated response to infection is broadly understood to involve recruitment to the airways of high numbers of innate and adaptive immune cells from a variety of subpopulations. Pro-inflammatory products released from these cells following their activation are held to be responsible for the acute damage to local mucosal tissues that triggers the symptoms characteristic of this disease [1]. Key aspects of the underlying activation process, and the full range of its cellular targets, are incompletely understood. An important question that remains to be resolved concerns what signals are released from the airways and how these impact the circulating immune cells prior to their migration into the airways. Such signals may be suitable targets for therapeutic development efforts.

There is compelling evidence from experimental murine asthma [2], and human atopic asthma [3] in which “exacerbations” are elicited by bronchial allergen challenge, that activation occurs in immune cells prior to their migration towards the airways. One such example is lung homing eosinophils that are activated prior to their release from bone marrow [3]. The extent to which similar processes occur in other cell populations involved in asthma pathogenesis, and the applicability of this general paradigm to “natural” (including viral triggered) asthma exacerbations, remains to be established.

In the present study, we have investigated systemic processes activated during an asthma exacerbation employing a genomics-based systems level approach involving comparative cellular and transcriptomic profiling of peripheral blood mononuclear cells (PBMC) collected from asthmatic children presenting to hospital emergency during an acute exacerbation, versus a second “resting” sample collected subsequently from each participant, at a time remote from the exacerbation. Flow cytometry and bulk RNA-Sequencing (RNA-Seq) combined with network analysis was employed to elucidate the signals impacting the circulating immune cells during acute asthma, by comparing PBMC response patterns between acute and convalescent visits. Our findings suggest that circulating inflammatory cells, preceding their homing to the airways, demonstrate a multitude of pre-programmed asthma-associated gene networks involving myeloid cell activation and differentiation in combination with leukocyte migration signatures. The strongest upstream drivers of these signatures were bacterial lipopolysaccharide (LPS), glucocorticoids and the master regulator of immune responses transforming growth factor beta 1 (TGFB1).

## Materials and methods

### Sample collection

The study population comprised school-age children (n = 19) presenting with an exacerbation of asthma to the Emergency Department at Princess Margaret Hospital for Children, Perth Australia [4]. Inclusion criteria were a gestational age of ≥ 36 weeks and with no known chronic underlying disorder, apart from asthma. Peripheral blood was collected from each participant during their acute exacerbation (acute visit = AV), on average 8.4 h after their most recent administration of systemic glucocorticoid treatment (standard dose of 1 mg/kg of patient’s weight), and at follow-up ≥ 3.9 weeks later (convalescent visit = CV), when the children were clinically stable and had no symptoms. Peripheral blood mononuclear cells (PBMC) were cryobanked as previously described [5]. Following thawing, we recovered > 90% of viable PBMC. We have previously shown that relevant gene expression is not affected by cryopreservation when assessed by real-time quantitative reverse transcription [6].

### Atopic status determination

Atopic status was defined as house dust mite (HDM) specific IgE ≥0.35kU/L or total IgE ≥300kU/L and/or skin prick positive HDM and/or cat at the acute visit. Serum total IgE, inhalant allergen (house dust mite) specific IgE, and cat specific IgE were quantified as previously described at the acute visit [5]. Skin prick tests were completed using house dust mite and cat allergen (wheal sizes ≥3 mm = positive) [5]. Asthma was diagnosed by the treating physician at presentation to the Emergency Department.

### Viral detection

Nasal specimens collected during the acute exacerbation were analysed for respiratory viruses as previously described [4, 7].

### Flow cytometry

Immunostaining of viable PBMC was conducted employing a panel of 12 monoclonal antibodies and appropriate isotype control as previously described [8, 9]. Sample compensation, quality control and manual gating was carried out with FlowJo software (version 10.0.8r1) as detailed in the Additional Material and Additional Fig. 1/Additional Table 1. Flow cytometry standard (fcs) samples were compensated and down-sampled fcs files (50,000 events per sample) were exported from FlowJo and imported into R 4.1.1 using *FlowCore* [10]. Flow-SOM clustering, dimensionality reduction, cluster visualisation (UMAP) and annotation were carried out using *CATALYST* [11]. Comparison of differential abundance between clusters was assessed using *diffcyt* [12].

### Plasma proteins

Plasma was separated from blood and stored at -80 °C until further processing. Endotoxin was quantified with the Limulus amebocyte lysate (LAL) assay (Hycult). ELISAs for soluble CD14 (sCD14; R&D Systems), Neopterin (IBL International), fibronectin 1 (FN1; R&D Systems) and S100A8/S19 (R&D Systems) were carried out following the manufacturer’s instructions.

### Statistical analysis

Population data that were non-normally distributed were presented as medians and interquartile ranges. Differences in sample ranks of cellular frequencies were assessed with a Wilcoxon test for paired comparisons and a *P-value* ≤ 0.05 was considered statistically significant. Absolute cell numbers (per ml of blood) are presented as ratios (acute: convalescent; mean ± Standard Error of the Mean (SEM)), and relative proportions (frequencies) are presented as median ± Standard Deviation (SD).

### RNA isolation and bulk RNA-Sequencing (RNA-Seq)

Total RNA extraction, integrity assessment, and subsequent sequencing employed standard methodology as detailed in the Additional Material.

### Differential expression analysis

Raw sequencing reads were aligned to the reference genome (hg19) and summarised as gene-level counts (see Additional Material). Raw read counts were filtered by retaining counts with at least one read per gene. Differentially expressed genes were identified employing the DESeq2 package [13] with False Discovery Rate (FDR) correction for multiple comparisons [14]. DESeq2 is based on a negative binomial distribution and an additive linear model is fit to the data. Samples are contrasted in a paired design comparing acute to convalescence for each participant. DESeq2 differential expression analysis was performed in conjunction with Remove Unwanted Variation (RUV) [15], to adjust the analysis for latent variation (see Additional Material).

### Upstream regulator analysis

Upstream regulator analysis (www.ingenuity.com) was employed to identify putative molecular drivers of the observed differentially expressed genes, as detailed previously [16, 17].

### Pathways analysis

Comprehensive gene set enrichment/pathways analysis was carried out separately for up and down regulated genes employing InnateDB [18].

### Network analysis

To obtain a holistic understanding of the gene expression program we employed weighted gene co-expression network analysis (WGCNA) as detailed in the Additional Material. Exacerbation-associated coexpression modules were identified employing module eigengenes, summarising the overall expression of each module based on the first principal component. Gene network modules were reconstructed employing Network Analyst [18]. For this, the network was constructed using integrated (union) protein-protein (IMEx Interactome DB, zero order network) and transcription factor-gene interactions (JASPAR database), to form a single reconstructed wiring diagram. Edges were filtered by degree to include < 1000 edges and visualised in Cytoscape [19] (version 3.6.1).

### Microarray data analysis

We downloaded a microarray dataset (accession no. GSE16032) [6], from the Gene Expression Omnibus, consisting of n = 3 pooled PBMC obtained from n = 25 atopic asthmatic children (age: 7.3 ± 0.7 years, mean ± SEM; 95% atopic; 85% virally infected; 95% of virally infected were rhinovirus positive) sampled at acute exacerbation in hospital emergency and at follow-up when well. Raw data quality control, filtering and annotation of probe sets is described in detail in the Additional Material. Differentially expressed genes were identified with LIMMA [20] with FDR adjusted *P-values*.

## Results

### Asthma exacerbation is associated with depletion of circulating immune cells

To assess the immunological response to an acute exacerbation of asthma, we assessed the abundance of circulating immune cells in the PBMC population from 19 children with MD-diagnosed atopic asthma as they presented to the Emergency Department at Princess Margaret Hospital for Children in Perth, Western Australia. The characteristics of the study population are presented in Table 1.

**Table 1:** Characteristics of the study population during acute asthma when the study participants presented to hospital

	Atopic asthmatic
**Number of participants**	19
**Male**, n (%)	12 (63)
**Age at recruitment in years**, median (range)	8.7 (6.9–13.2)
**Inhalant allergy positive** (%)	100^a^
**HDM-specific IgE (kU/L)**, median [interquartile range]	8.3 [1.7–33.4]
**Cat IgE (kU/L)**, median [interquartile range]	0.5 [0.2–6.8]
**Total IgE (kU/L)**, median [interquartile range]	585.3 [241.6–926.0]
**Rhinovirus (HRV)**, prevalence, positive/tested, (%)	11/14 (78.6)^b^
**Respiratory syncytial virus (RSV)**, prevalence, positive/tested, (%)	0/13 (0.0)
**Adenovirus (AdV)**, prevalence, positive/tested, (%)	0/13 (0.0)
**Influenza virus (InfV)**, prevalence, positive/tested, (%)	1/13 (7.7)
**Parainfluenza virus (PIV)**, prevalence, positive/tested, (%)	1/13 (7.7)

^a^Inhalant positive 18/19 based on IgE titre; 1 participant based on skin prick test (SPT). Virology data was only available for ^b^14 participants. Skin prick test were classified positive if wheal diameter was ≥ 3 mm

**Fig. 1:**
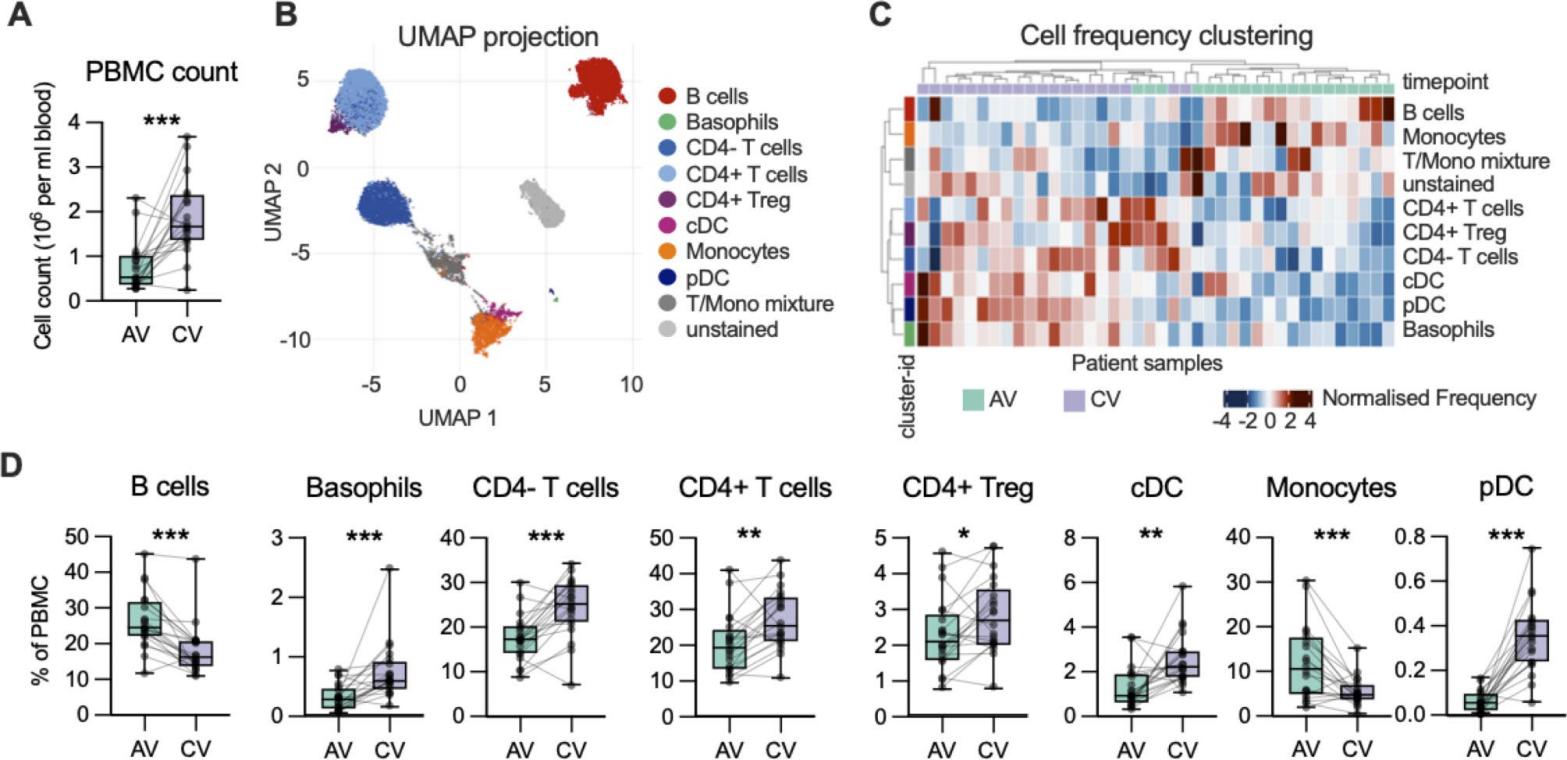
**Inflammatory cells are trafficking and leaving the peripheral blood**. (**A**) Number of PBMC per ml of blood during the acute (AV) and convalenscent (CV) timepoint. (**B**, **C**) Visualisation or FlowSOM clusters using UMAP (**B**) and clustering of immune cell subset abundance for each individual in (**C**). (**D**) Frequency of specific immune cell subset cluster for each individual compared at the two timepoints. Significance of differences are calculated using Wilcoxon test for paired analysis and displayed as: ***, < 0.001; **, < 0.01 and *, < 0.05

All study participants were inhalant allergy positive (see atopy definition in Methods) and 78.6% tested positive for rhinovirus. One participant tested positive for influenza virus and one for parainfluenza virus (both also testing positive for rhinovirus). There was no correlation between time since systemic glucocorticoid treatment and either IgE levels or SPT outcomes (Additional Fig. 2). A marked reduction of total PBMC counts was observed during the acute exacerbation compared to convalescence (Fig. 1A). For analysis of changes in specific immune cell subset abundance, FlowSOM clustering was used to identify cells with a similar marker expression profile. These were annotated and visualised using UMAP (Fig. 1B). By clustering immune cell abundance, the samples split across the two timepoints (Fig. 1C). Indeed, when comparing abundance of the annotated clusters, significant changes in abundance was observed for all subsets with an increase in proportion during the acute phase observed for B cells and monocytes, whereas a decrease was observed for basophils, T cells and dendritic cell subsets (Fig. 1D), suggesting that these latter subsets may migrate into the airways during an exacerbation. The most dramatic depletion was observed for plasmacytoid dendritic cells, which are potent producers of type I interferons responses during rhinovirus infections [21] (Fig. 1D). Comparable results were also observed using manually gated data (Additional Fig. 3A-B). Time since administration of systemic glucocorticoids was positively correlated with abundance of cDC and monocytes but not other immune cell populations during the acute event (Additional Fig. 4).

**Fig. 2:**
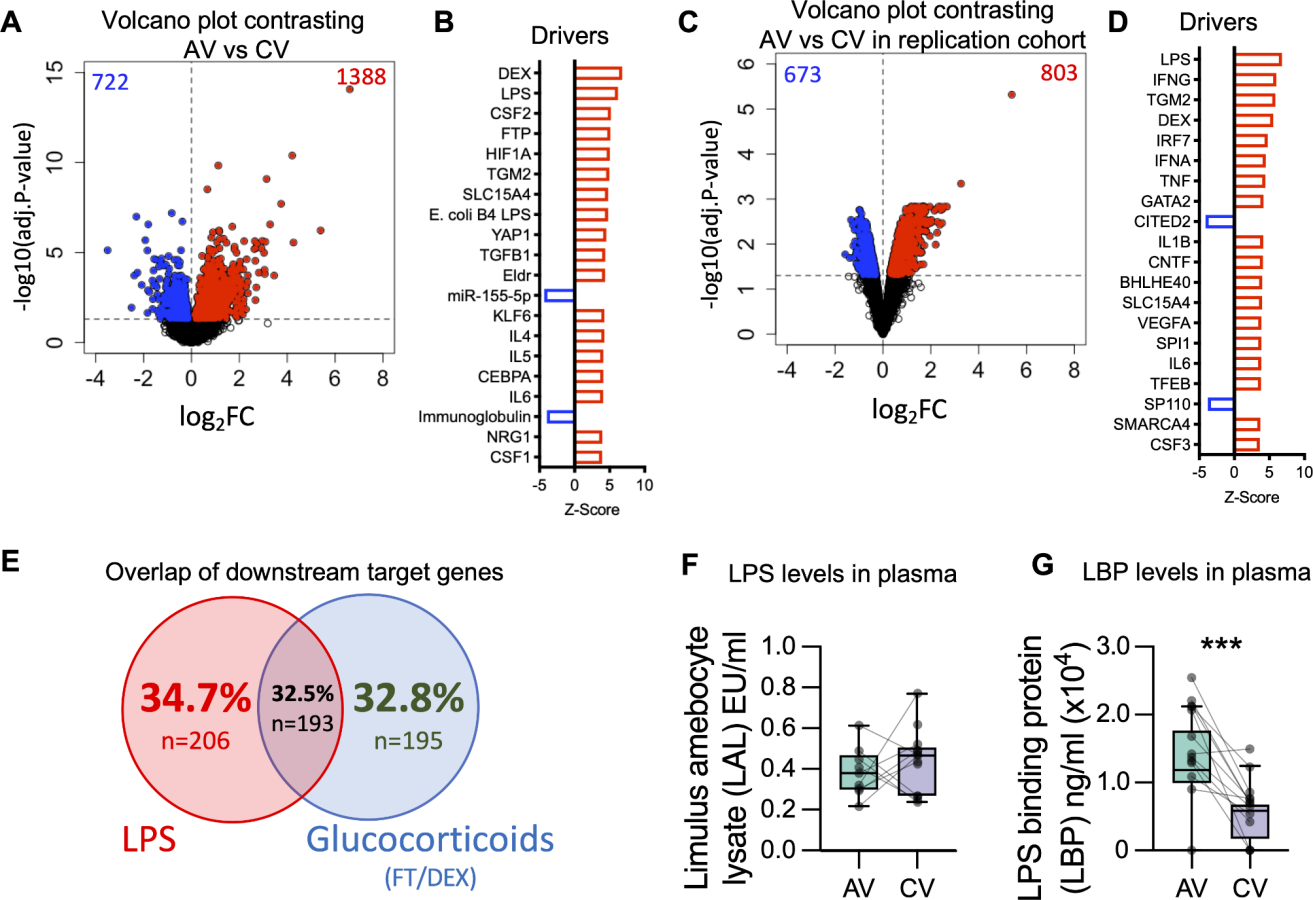
**Identification of differentially expressed genes and putative molecular drivers.** Differentially expressed genes were identified comparing acute exacerbation versus convalescence in (**A**) with DESeq2 analysis and (**B**) LIMMA in independent samples (GSE16032). (**A/C**) Volcano plots showing the –log10(adjusted P-value) plotted against the log_2_ fold change, in (**A**) atopic asthmatic children (n = 19), and in (**C**) independent samples (GSE16032) consisting of atopic asthmatic children (n = 25). Upregulated genes = red. (**B/D**) Top molecular drivers identified employing the Ingenuity Knowledge Base. Red = activated driver, blue = inhibited driver. Absolute activation *Z-scores* ≥ 2.0 and *P-values* ≥ 0.01 were deemed significant. FTP = fluticasone propionate, DEX = dexamethasone. (**E**) Venn diagram illustrating the overlap of target genes from the drivers LPS, TGFB1 and glucocorticoid. (**F**) LPS was measured with limulus amebocyte lysate (LAL) assay (Endotoxin Unit = EU) and (**G**) the LPS binding protein (LPB) was increased in plasma at AV relative to CV. The *P-value* is derived from a Wilcoxon test for paired analysis, ***,<0.001

**Fig. 3:**
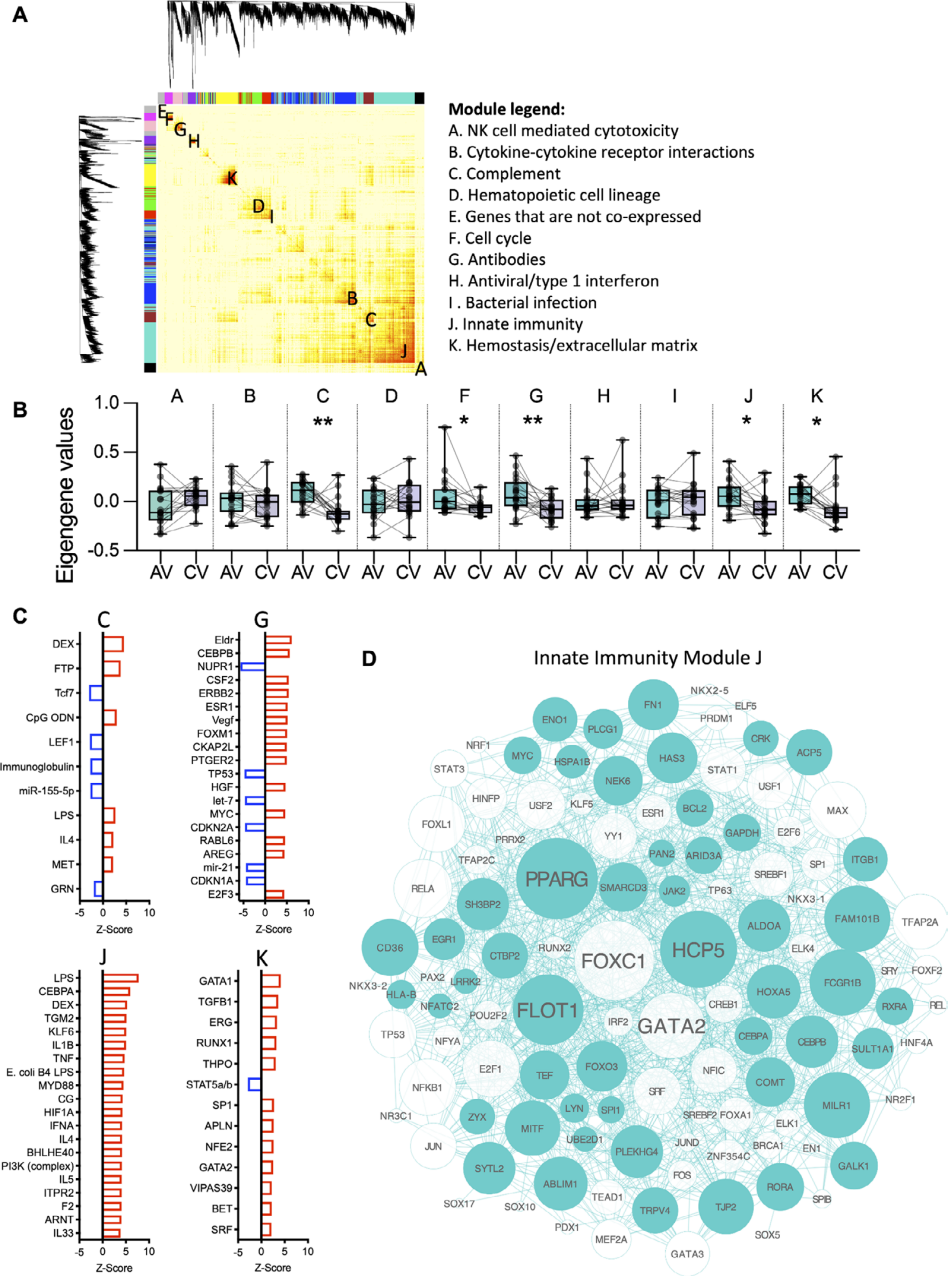
**Network analysis reveals five exacerbation-associated gene modules.** Network analysis (WGCNA) was performed utilising the top ~ 4000 most variable genes, in (**A**) heatmap of the gene networks with hierarchical clustering gene dendrograms where each column and row represent single genes. Red colour indicates highly co-expressed clusters of genes or gene networks across the diagonal, and lighter colour in white/yellow indicates low co-expression, in (**A**, **B**) the data clustered into 11 co-expression modules denoted modules A-K, and the overall expression of the modules was determined using eigengene values contrasting acute exacerbation versus convalescence. Wilcoxon signed ranked test was used to test for differences between the acute and convalescent visits (AV/CV). *,<0.05, **,<0.01. In (**C**) putative molecular drivers were identified for the significant modules with Ingenuity Pathways Analysis from the Ingenuity Knowledge Base. Red = activated driver, blue inhibited driver. Absolute activation *Z-scores* ≥ 2.0 and *P-values* ≥ 0.01 were deemed significant. FTP = fluticasone propionate, DEX = dexamethasone, CpG ODN = CpG oligodeoxynucleotide. (**D**) Reconstruction of the wiring diagram of the innate immunity module J and visualisation using Cytoscape. Coloured node = present in the original network (zero order network), white node = transcription factors

### Acute asthma exacerbations induce transcriptional changes in PBMC

To assess transcriptional changes to the gene expression program during asthma exacerbations, PBMC were profiled by RNA-Seq. Comparison of gene expression profiles in PBMC between acute exacerbation and convalescent visits identified 2,160 differentially expressed genes; 1,388 genes were upregulated and 772 downregulated (false discovery rate (FDR) < 0.05; Fig. 2A, Additional Table 2). Of those, the IL1 receptor *IL1R2* (adj.P = 4.11 × 10^− 11^), *IL13RA1* (adj.P = 3.64 × 10^− 3^) and *CCR2* (adj.P = 1.05 × 10^− 3^) were among the differentially expressed genes where *CCR2* is known for its lung homing function (Additional Table 2). Pathways analysis demonstrated that the upregulated genes were enriched for hemostasis (p = 3.64 × 10^− 13^), antibody-mediated complement activation (p = 3.38 × 10^− 7^), Fcgamma receptor (FCGR) dependent phagocytosis (p = 8.31 × 10^− 7^), EGFR1 signalling (p = 2.53 × 10^− 5^), endogenous TLR signalling (p = 1.40 × 10^− 3^) and B cell receptor signalling (p = 1.62 × 10^− 3^). Downregulated genes were enriched for components of the translation machinery (p = 7.12 × 10^− 6^), IL-12-mediated signalling (p = 2.63 × 10^− 4^) and T cell receptor signalling (p = 3.81 × 10^− 2^). To identify putative molecular drivers of the differential gene expression patterns the data were further interrogated using upstream regulator analysis (Fig. 2B). The top-ranking activated drivers were glucocorticoids (dexamethasone (DEX), fluticasone propionate (FTP)), lipopolysaccharide (LPS)/E.coli B4 LPS, colony stimulating factor 2 (CSF2), proliferation associated genes hypoxia-inducible factor 1 alpha (HIF1A), EGFR long non-coding downstream RNA (ELDR), yes-associated protein 1 (YAP1) and transforming growth factor beta 1 (TGFB1), Th2 cytokines interleukin (IL)-4 and IL-5, as well as the pro-inflammatory enzyme transglutaminase 2 (TGM2) and cytokine IL-6. To confirm the findings in an independent cohort, we utlised a microarray dataset from a study we previously published (GSE16032)[6], comparing transcriptomic profiles of acute exacerbation and convalescent PBMC samples from asthmatic children. Reanalysis of these data identified 1,476 genes differentially expressed at exacerbation (Fig. 2C), and upstream regulator analysis revealed a list of molecular drivers (Fig. 2D) that included 5 of the top 20 drivers (DEX, LPS, TGM2, IL6, SLC15A5) in the data set from the present study. Of note, type 1 interferon pathways (IRF7, IFNA) featured in the top drivers in the replication cohort, which had a 95% incidence rate of rhinovirus infection and similar age range (see Methods), whilst in the current study, IFNA did not meet the significance threshold (z-score = 1.84). The regulator analysis from the two independent cohorts hence suggests that the drivers LPS, and glucocorticoids (DEX/FTP) are likely relevant drivers of the transcriptional program observed during asthma exacerbations in atopic children.

To further explore the overlap, downstream targets of these regulators were identified. The data showed that there were 193 (32.5%) common targets between the drivers LPS and glucocorticoids (Fig. 2E), LPS had the larger number of unique downstream targets (n = 206, 34.7%) compared to glucocorticoids (n = 195, 32.8%). As LPS was identified as the major driver of the transcriptomic profile observed during asthma exacerbation, we assessed plasma levels of endotoxin/LPS and LPS associated proteins. Although there was no difference in LPS levels (measured by limulus amebocyte lysate assay, Fig. 2F), LPS binding protein (LBP) was significantly increased during the acute exacerbation compared with convalesence (Fig. 2G). No changes in plasma concentration of sCD14, neopterin, fibronectin 1 (FN1) and S100A8/S100A9 were observed (Additional Fig. 5). Given that LPB markedly enhances the sensitivity of myeloid cells to LPS [22], the data suggests that LPS responses might be exaggerated during acute asthma exacerbations.

In our study the participants were treated with steroids, and corticosteroids are known to have an impact on the gene expression in PBMC, we therefore computed eigengene values (summarising the first principal component) from the LPS and DEX/FTP signatures, and correlated these with the time since steroid treatment. The data showed that the time since steroid administration did not correlate with either the LPS nor the DEX/FTP signatures (Additional Fig. 6).

### Network analysis reveals five acute exacerbation-associated gene modules

To obtain a systems level understanding of the exacerbation responses, we constructed a coexpression network using WGCNA employing the most variable genes of the transcriptomic data derived from the PBMC samples across both visits (Fig. 3A). The resulting network contained 4,679 genes, organised into 11 co-expression modules (labelled A-K; Fig. 3A-B). To summarise the overall expression of each gene module, eigengene values were calculated and compared between the two visits. The data showed that five modules, modules C, F, G, J and K were significantly perturbed at the acute visit compared to the convalescent visit (Fig. 3B).

To probe the biological functions associated with the gene network modules, pathways analysis was performed using InnateDB (Additional Table 3). This analysis suggested that the five disease-associated modules were enriched with the following pathways: Module C - the complement pathway; Module F - the cell cycle; Module G - antibody-mediated complement activation and B cell receptor signalling; Module J – innate immunity; and Module K - hemostasis/ extracellular receptor interactions. Of note, there was also an antiviral/type 1 interferon module H, however, this module was not differentially perturbed between visits.

To identify module driver genes we employed upstream regulator analysis (Fig. 3C). The data showed that the top-ranking drivers, identified in the differential expression analysis, namely LPS, TGM2, TGFB1, IL-4/IL-5, and the glucocorticoids, partitioned to some extent into separate modules. Key activated drivers of module C, the complement module, were: glucocorticoids, LPS, CpG oligodeoxynucleotides (CpG ODN) and IL-4. Module F only contained one significant driver: methylprednisolone. Module G, the antibody module, was putatively driven by proliferation/growth factors including ELDR, ERBB2, VEGF and HGF and pro-inflammatory CEBPB. The top drivers of Module J, the innate immunity module: LPS, DEX, inflammatory CEBPA, IL1B, TNF, as well as type 1 interferon (IFNA, MYD88), and finally Module K, the ECM module: the top regulators included the transcription factors GATA binding protein 1 (GATA1) and TGFB1 (Fig. 3C).

The network wiring diagrams of the significant modules (C, F, G, J, K) were reconstructed using Network Analyst, applying a zero order network of literature-curated protein-protein interactions and transcription factors (Fig. 3D, Additional Fig. 7). The innate immunity module J, that is predicted to be regulated by LPS is shown in Fig. 3D. Overall, the data showed common transcription factors that target all the network modules including GATA-binding factor 2 (GATA2), Forkhead Box C1 (FOXC1), Yin Yang 1 (YY1) and Forkhead Box L1 (FOXL1).

Given that PBMC comprise a heterogeneous cell population, and variations in cellular composition may potentially confound the analyses, we repeated our analyses with or without adjustment for cellular proportions of monocytes, T and B cells. The differential expression analysis results were overlaid on a module-by-module basis and the only substantive effect observed was when adjusting for monocyte numbers, and as a result, modules C (adjusted p = 0.07) and K (adjusted p = 0.12) were no longer differentially expressed (Additional Fig. 8), suggesting that expression of these network modules was monocyte-associated.

## Discussion

A feature of the acute phase of asthma exacerbations is the rapid migration of immune cell populations into airways tissues, resulting in transient reduction in circulating numbers of PBMC including T-cells and dendritic cell populations with less impact observed on monocyte and B cell populations. Monocytes are recognised to be an important component of the overall cellular infiltrate that accumulates in the airways following exacerbation [23], and the difference observed here relative to other myeloid (and lymphoid) populations may reflect variations in underlying migration kinetics, or in bone marrow output. Hematopoietic stem and progenitor cells in the bone marrow are known to act as sensors for peripheral inflammation/infection and will increase the proliferation of myeloid lineage cells that are released into system circulation to deal with environmental stimuli [24].

A key finding in this study was the demonstration of a complex pre-programmed coexpression network operative within PBMC circulating at the time of acute asthma. The genes in this network were organised into a range of immunoinflammatory pathways, in particular those associated with activation of innate immune and inflammatory function(s), cell migration and tissue homing, and antibody-mediated complement activation. Principal to this, was the demonstration that the top drivers from the differential expression analysis (LPS, glucocorticoids) were partitioned into respective modules J, C, G and K, and corresponding to unique biological expression signatures. Given that the children were treated with steroids, which could potentially impact on cellular immune responses and gene expression profiles, we investigated the relationship between time since administration of systemic glucocorticoids and ensuing cellular and molecular responses. We found that the time since administration of steroids was positively correlated with the abundance of cDC and monoctes, but there was no relationship with either the LPS nor DEX/FTP signatures, and therefore the LPS signature could not be explained by the steroid treatment. Analyses adjusting for monocytes identified myeloid cells as the source of modules C and K, and this conclusion is consistent with our earlier studies on PBMC samples from exacerbating children, employing cell sorting in conjunction with RT-qPCR profiling [6].

LPS-driven activation signatures are prominent at the acute exacerbation timepoint, notably LPS appears as a significant driver associated positively with downstream genes in myeloid-associated module C (Fig. 3). Interestingly, no change in circulating LPS levels was observed, thus this does not support the ‘leaky gut’ theory of dysfunctional gut mucosal barrier during respiratory infections leading to circulating LPS. In contrast, we observed an increase in LPS binding protein, which markedly enhances the sensitivity of myeloid cells to LPS [22], detected at the acute timepoint relative to convalescense. Enhanced sensitivity to LPS is likely to modulate the severity of acute asthma exacerbations through exposure to environmental LPS [25] and/or the presence of pathogenic gram negative bacteria in the airway microbiome [26].

This LPS signature is consistent with contributions from secondary bacterial infections as the exacerbation response progresses, as inferred from earlier studies [26–29], and/or a marker for endogenous TLR signalling [25]. Moreover, McCauley and colleugaues recently demonstrated interactions between viruses and bacteria in children with high-risk for exacerbations, showing that bacterial family members *Moraxella* and *Haemophilus* are enriched in nasal specimen of children that test positive for a respiratory virus and progress to experience exacerbations [30]. Furthermore, our group has recently shown that in vitro LPS stimulation of PBMC from children at high-risk of developing asthma are associated with signalling plasticity and immature type 1 interferon signalling gene networks [31], which can be modulated by in vivo treatment of a bacterial lysate mixture that in turn reduces the risk of lower respiratory tract infections [32].

In relation to cell trafficking-associated signatures, we additionally surveyed exacerbation-associated gene expression profiles for the activation status of specific chemokine receptor genes, and it was noteworthy that the receptor gene exhibiting a high level of expression was *CCR2*, which in other studies has been strongly associated with lung homing [6, 33]. These findings are generally consistent with a model in which signals generated as part of the exacerbation process are sensed by myeloid precursors in bone marrow, thus altering the programming of immature emigrants prior to their release into the blood and subsequent migration to the exacerbation site [34].

The precedent for the operation of such a systemic mechanism during asthma exacerbations are studies in mouse and humans relating to pre-activation of eosinophil precursors in bone marrow following bronchial challenge of sensitised atopics with aeroallergen. In that experimental system, IL-5-secreting aeroallergen-specific Th2 cells are initially activated in the airways and then migrate to bone marrow where they mediate local IL-5-driven pre-activation of eosinophils, which then traffic back to the lung to mediate the eosinophil-driven asthma late phase response [35].

Upregulated cell cycling module F (Fig. 3B) is consistent with the enhanced release into the circulation of immature PBMC subpopulations triggered by this viral-initiated exacerbation event. In this regard, it is notable that at this stage of the exacerbation cycle antiviral signatures are present within the overall coexpression network (within module H in Fig. 3B), but they were not consistently differentially expressed across all participants. The attenuation of the antiviral signatures in a large subset of the participants at this sampling time may reflect the preferential depletion of circulating pDC which are the primary source of type 1 interferons, possibly reflecting the rapid trafficking of these protective cells, which constitute the line-of-defence against viruses, to the infection site [36]. Alternatively, antiviral responses may be deficient in a subset of the children as suggested from previous studies [37–39], or the observed heterogeneity may also be due to the time of collection relative to onset of infections, since anti-viral interferon responses peak within 48 h [38].

The most significantly upregulated network modules C and G are regulated by glucocorticoids and growth factors, respectively. The presence of mainly immunoglobulin-related gene signatures in the module G may reflect the transient boosting of IgE production which is a notable component of the exacerbation response [6, 40]. The strong upregulation of glucocorticoid-associated module C is likely a direct result of the high dose steroid treatment (standard dose of 1 mg/kg of child’s weight), given to all participants upon hospital presentation with acute asthma (on average 7.7 h prior to blood collection), potentially with additional effects from the endogenous hormone. Glucocorticoids are known to inhibit pro-inflammatory pathways and adaptive immunity, whilst preserving innate mechanisms in airway epithelial cells [41]. It is noteworthy that this module contained the upregulated C1Q complex (Additional Table 3), which is the first component of the classical complement cascade and the prototypical innate anti-microbial defence mechanism, its functions including promotion of opsonisation/phagocytosis, lysis of bacteria/infected cells, augmentation of antibody responses [42], but also inhibition of Th2-associated airways eosinophilia [43] and IFN mediated signalling in pDC [44].

Of interest in this study was the identification, within the co-expression network, of the immunomodulatory cytokine TGFB1 amongst the most dominant drivers of the myeloid-associated module K (Fig. 3C). TGFB1 is a highly pleiotropic cytokine with myriad functions that are likely to be location specific. There is significant literature associating genetic/epigenetic variations in TGFB1 and its downstream signalling pathway (SMAD3) to asthma [45–47] and atopy [48] and heightened susceptibility of allergic disease [49]. Our group has recently demonstrated, utilising a dichotomous system of high versus low risk atopy/non-atopic susceptibility to asthma (BN/PVG strain rats), that co-exposure to aeroallergen (ovalbulmin) and rhinovirus infection (rodent equivalent = attenuated mengovirus), resulted in exaggerated transcriptional profiles in susceptible lungs and regulation of pathways by TGFB1/SMAD3 signal was identified in high risk animals [50]. TGFB1 has also been implicated in tissue remodelling (i.e. fibrosis) of the lung parenchyma and airways in asthmatics [51, 52], and various studies have shown elevated expression of TGFB1 in BAL and biopsies [53, 54], and also in serum from asthmatic participants [55]. In the airways, the main source of TGFB1 is thought to be from eosinophils [56], and depletion of eosinophils in asthmatics, employing anti-IL-5, results in decreased TGFB1, suggesting eosinophils are important contributors to airways remodelling [57]. The presence of TGFB1 as a major upstream driver of the exacerbation-associated networks suggests that it has also been expressed and had its effects on myeloid precursor populations in the bone marrow prior to their release into the blood. Several studies have also shown that TGFB1 potentiates replication of rhinovirus by suppressing key antiviral genes, type I and III interferons and inducing cell cycle arrest [58–60]. This is of relevance as rhinovirus infection was confirmed in 78.6% of study participants at the acute event. It has been demonstrated that viral infection in vitro impairs the action of glucocorticoids, independent of virus type [61], which emphasises the need for novel treatment options since the standard treatment for asthma exacerbations involves glucocorticoid therapy [62].

Our study has limitations that should be acknowledged. The gene profiling was based on PBMC, a heterogeneous cell population, and hence differential gene expression signatures observed may be influenced by changes in the cellular composition, although these were controlled for statistically. We cannot discern the transitional stage at which we sampled the PMBC; some PBMC could be primed for migration to the tissues, whilst others may be emerging from the bone marrow, and some PBMC may be recirculating from inflamed tissues and yet other cells could simply remain in circulation. Molecular profiling on sorted cell populations or single cell RNA-Seq would overcome this limitation. In addition, tracing studies labelling bone marrow or PBMC could be carried out in further studies. Furthermore, no control samples from healthy children with or without a viral infection were available. Nevertheless, acute samples were paired with convalescent samples providing internal controls for each participant. Lastly, the study participants were recruited in the Emergency Department with severe illness, providing mechanistic insights into real-world immune activation. However, as a consequence, all of our participants were treated with steroids before blood was collected and therefore, the clinical assessments, cellular immune and molecular responses, cellular immune and molecular responses were performed post-steroid treatment and may have been impacted by steroids. To address this, we evaluated the impact of steroid treatment and found no significant relationship between total serum IgE and atopy with the time since steroid administration. With regards to cellular and molecular responses, whilst time since steroid treatment was positively correlated with the abundance of cDC and monocytes, there was no significant relationship with the LPS activation signature.

In summary, this study demonstrates considerable changes in circulating immune cell subset abundance during an acute asthma exacerbation, which is accompanied by differential gene expression and modulation of signalling networks, some of which are driven by LPS/LBP, glucocorticoids and TGFB1. This study advances our understanding of underlying disease mechanisms in asthma and provides a new approach for identification of targets for mechanistic studies and drug repurposing programs. Moreover, we demonstrate that components of the inflammatory cell activation process, associated with asthma exacerbations, are triggered systemically, prior to their recruitment into the lung, which would enable therapeutic targeting of relevant precursor cell populations systemically as opposed to only after their recruitment to the airways, as a valid approach towards improved asthma control. Importantly, the demonstration of increased systemic LPS binding protein during the acute exacerbation phase, may enhance the sensitivity to LPS and is likely to modulate the severity of acute asthma exacerbations in children.

## Electronic supplementary material

Below is the link to the electronic supplementary material.

**Additional file 1**: Extended Methods

**Additional file 2: Fig. 1**. Exemplary gating strategy for all subsets analysed. Example gating strategy from acute peripheral blood mononuclear cells labelled with a panel of antibodies to identify lymphoid and myeloid cells subsets

**Additional file 3: Fig. 2**. Correlation with Atopy readouts and systemic glucocorticoid treatment. Total serum IgE and SPT cumulative wheal size did not corelate with time since administration of systemic glucocorticoids

**Additional file 4: Fig. 3**. Inflammatory cells are trafficking and leaving the peripheral blood. Peripheral blood mononuclear cells (PBMC) were sampled from atopic asthmatics at presentation to hospital Emergency during an exacerbation, the acute visit (AV), and following recovery at the convalescent visit (CV). Multi-colour flow cytometry was employed to quantify inflammatory cell subsets, in (A) ratio of acute: convalescent (cells per ml blood), data are represented as mean ± SEM, and (B) cellular frequency (percentage) of PBMC. The *P-values* are derived from a Wilcoxon test for paired analysis. ****,<0.0001, ***,<0.001, **,<0.01, *,<0.05

**Additional file 5: Fig. 4**. Correlation with cell subset abundance during the acute event and systemic glucocorticoid treatment. Abundance of immune cell subsets correlated with time since administration of systemic glucocorticoids. Correlation was assessed using Pearson’s parametric correlation on samples for which data on steroid treatment was available, N = 18, **, p < 0.01, *, *p* < 0.05

**Additional file 6: Fig. 5**. Plasma levels measured of soluble CD14 (sCD14), neopterin, fibronectin 1 (FN1) and S100A8/S19

**Additional file 7: Fig. 6**. Correlation of LPS and steroid module eigengene signatures from the upstream regulator analysis during the acute event and time since glucocorticoid treatment. Time since steroid administration does not correlate with either the DEX/FT or the LPS signature. Correlation was assessed using Pearson’s parametric correlation, n.s. = not significant

**Additional file 8: Fig. 7**. Reconstruction of the wiring diagram of modules C, F, G and K. Coloured node = present in the original network (zero order network), white node = transcription factors

**Additional file 9: Fig. 8**. Identification of exacerbation-associated modules with or without adjustment for cellular composition. The data was analysed with DESeq2/RUVSeq and adjusted for cellular composition, in (a) unadjusted networks, (b) adjustment for proportions of monocytes, (c) adjustment for proportions of B cells, and (d) adjustment for proportions of T cells. The dashed horizontal line indicates an adjusted p-value < 0.05. **median adjusted P-value < 0.01, *median adjusted P-value < 0.05, ns = not significant

**Additional file 10: Table 1**. Multi-parameter flow cytometry panel. **Table 2**. Differentially expressed genes comparing acute versus convalescent visits. **Table 3**. Pathways analysis for modules A-K

## Data Availability

The datasets supporting the conclusions of this article are available in the Gene Expression Omnibus repository [GSE96530, GSE16032; http://www.ncbi.nlm.nih.gov/geo/]. Supplementary methodology is supplied in the Additional Material.

## Abbreviations

AV: Acute visit
CV: Convalescent visit
FDR: False discovery rate
GC: Glucocorticoids
IgE: Immunoglobulin E
LPS: Lipopolysaccharide
PBMC: Peripheral blood mononuclear cells
RNA-Seq: RNA Sequencing
TGFB1: Transforming growth factor beta 1
WGNCA: Weighted gene network co-expression analysis
