# Supplementary material for: LPS binding protein and activation signatures are upregulated during asthma exacerbations in children
Source: Respir Res. 2023 Jul 12;24:184. doi: 10.1186/s12931-023-02478-3 (PMC10337076; doi:10.1186/s12931-023-02478-3)
Supplement: Supplementary file 1 — Additional file 1: Extended Methods [file 12931_2023_2478_MOESM1_ESM.docx]

***Additional Material***

**LPS binding protein and activation signatures are upregulated during asthma exacerbations in children**

**Authors**

Anya C Jones, PhD^1,2^, Jonatan Leffler, PhD^1^, Ingrid A Laing, PhD^1,3^, Joelene Bizzintino, PhD^1,3^, Siew-Kim Khoo, BSc^1,3^, Peter N LeSouef, MD^2^, Peter D Sly, MD DSc^4^, Patrick G Holt, DSc FAA^1,4^, Deborah H Strickland, PhD^1#^, Anthony Bosco, PhD^5, 6#^

**Affiliations**

^1^ Wal-yan Respiratory Research Centre, Telethon Kids Institute, University of Western Australia, Perth, Australia

^2^ UWA Medical School, University of Western Australia, Australia

^3^ Division of Cardiovascular and Respiratory Sciences, The University of Western Australia, Australia

^4^ Child Health Research Centre, The University of Queensland, Brisbane, Australia

^5^ Asthma and Airway Disease Research Center, University of Arizona, Tucson, AZ, United States

^6^ Department of Immunobiology, The University of Arizona College of Medicine, Tucson, AZ, United States

# These authors contributed equally to this work.

**Corresponding author**

A/Prof Anthony Bosco

Asthma & Airway Disease Research Center

The University of Arizona, The BIO5 Institute, Rm. 329, 1657 E. Helen Street, Tucson, AZ 85721

Phone: +1 (520) 604-3081; Email: abosco@arizona.edu

**Additional Methods**

**Flow cytometry**

Monoclonal antibodies were obtained from BD Biosciences. PBMCs were incubated for 30 minutes on ice with cocktails of extracellular markers: fluorochrome conjugated anti-human mABs CD19-FITC, CD4-V500, CD123-PE-CF594, CD11b-BV605, HLA-DR-PerCP, CD127-BV786, CD11c-PECy7, CD25-BV421, CD14-APC H7 and CD3-AF700. This staining was followed by 60 minutes intracellular staining with the mABs FcεRIα-APC and FOXP3-PE (eBiosciences). Isotype-matched control mAbs mIgG2bκ-APC and mIgG1κ-PE were utilised for setting negative gating. Single stains were employed for machine setup and the flow data was acquired on the LSRFortessa flow cytometer (BD Biosciences, San Jose, CA, USA).

**RNA isolation and RNA-Sequencing (RNA-Seq)**

Total RNA was extracted from viable PBMC employing TRizol (Ambion, Life Technologies) followed by RNeasy MinElute (Qiagen, Hilden, Germany). The integrity of the RNA was 9.5 ± 0.3 (mean ± SD), as assessed on the Bioanalyzer (Agilent Technologies, California, USA). Total RNA samples (500ng, n=38) were shipped to the Australian Genome Research Facility for library preparation (TruSeq Stranded mRNA Library Prep Kit) and sequencing (Illumina HiSeq2500, 50bp single-end reads, v4 chemistry). Up to 24 samples were multiplexed utilising unique barcode adaptor sequences ([1](#_ENREF_1)). Approximately twenty-four million reads were generated per sample. The raw sequencing data are available from GEO (accession no. GSE96530).

**Data pre-processing**

The RNA-Seq data were analysed in the R software environment for statistical computing. Raw sequencing reads were aligned to the reference genome (hg19) ([2](#_ENREF_2)) and summarised as gene-level counts employing summariseOverlaps ([3](#_ENREF_3)). Exploratory data analysis was carried out with EDASeq package ([4](#_ENREF_4)). Firstly, the raw read counts were transformed by plotting boxplots of the relative log expression (RLE), which tests log-ratio of each gene in each sample with the median across all samples. Secondly, a principal component analysis (PCA) was employed to identify patterns in the data by reducing the dimensionality of the data. Finally, RLE and PCA transformations were repeated following global-scale median normalisation to account for differences in sequencing depths, i.e. library size, across lanes.

RUVg normalisation was carried out utilising the 5000 least significantly differentially expressed genes from the DESeq2 analysis ([5](#_ENREF_5)) as ‘*in silico*’ empirical negative control genes to determine the factors of unwanted variation (*k*) in the data. The number of *k* was ascertained by plotting the RLE and PCA with increasing numbers of *k* and repeating the RUVg adjusted DESeq2 analysis by adding the estimated factors of unwanted variation to the linear model. The effect of differential cellular composition on the data was analysed in a single model.

**Upstream regulator analysis**

Upstream regulator analysis (www.ingenuity.com) was employed to identify putative molecular drivers of the observed differentially expressed genes ([6](#_ENREF_6)). IPA is based on prior knowledge of cause and effects between transcriptional regulators and target genes. Putative driver genes were deemed significant with absolute *Z-scores* ≥ 2.0 (positive *Z-scores* infer activation, negative scores indicate inhibition) and overlap Benjamini & Hochberg adjusted *p-values* ≤ 0.01. The overlap P-value is calculated using the right-tailed Fisher’s exact test to assess the overlap between regulators and the observed gene expression changes, i.e. it measures the over-presentation of regulator signatures.

**Pathways analysis**

Comprehensive gene set enrichment/pathways analysis was carried out separately for up and down regulated genes, employing InnateDB ([7](#_ENREF_7)). InnateDB encompasses > 196,000 molecular interactions and 3000 pathway annotations. The significance of overlap is calculated utilising hypergeometric distribution with a Benjamini & Hochberg correction for multiple testing.

**Network analysis**

To obtain a systems level holistic understanding of the gene expression patterns we employed weighted gene co-expression network analysis (WGCNA) ([8](#_ENREF_8), [9](#_ENREF_9)). The count data was transformed using the variance stabilising transformation algorithm from DESeq2. ComBat was employed to remove unwanted variation of the transformed data ([10](#_ENREF_10)). The DCGL package ([11](#_ENREF_11)) was utilised to identify the top ~4,000 most variable genes for constructing co-expression networks. The following parameter settings were employed: power = 7, Pearson correlation, minimum module size = 5, merge cut height = 0.1. Module eigengenes values were used to summarise the overall expression of each gene module based on the first principal component.

**Microarray analysis (independent samples)**

We downloaded a microarray dataset (accession no. GSE16032) ([12](#_ENREF_12)), from the Gene Expression Omnibus, consisting of PBMC obtained from atopic asthmatic children sampled at acute exacerbation in hospital emergency and at follow-up during convalescence. The quality of the raw data was assessed utilising the R package arrayQualityMetrics ([13](#_ENREF_13)). Raw expression values were pre-processed with the robust multi-array average (RMA) algorithm ([13](#_ENREF_13)), performing background correction, log2 transformation and quantile normalisation. A custom chip description file (hgu133plus2hsentrezgcdf, Version 20) was employed to annotate probe sets to genes based on updated genome and transcriptome information ([14](#_ENREF_14)). Principal component analysis-based filtering was employed (pvac package) and probe sets of low quality were removed ([15](#_ENREF_15)). Differentially expressed genes were identified with LIMMA ([16](#_ENREF_16)) with FDR adjusted *P-values*. Upstream regulator analysis (IPA) from the Ingenuity Knowledgebase was used to compare and confirm the list of putative molecular drivers of the gene expression patterns to the current dataset.

**References**

1. Shiroguchi K, Jia TZ, Sims PA, Xie XS. Digital RNA sequencing minimizes sequence-dependent bias and amplification noise with optimized single-molecule barcodes. Proc Natl Acad Sci U S A. 2012;109(4):1347-52.

2. Pertea M, Kim D, Pertea GM, Leek JT, Salzberg SL. Transcript-level expression analysis of RNA-seq experiments with HISAT, StringTie and Ballgown. Nature protocols. 2016;11(9):1650-67.

3. Anders S, McCarthy DJ, Chen Y, Okoniewski M, Smyth GK, Huber W, et al. Count-based differential expression analysis of RNA sequencing data using R and Bioconductor. Nature protocols. 2013;8(9):1765-86.

4. Risso D, Ngai J, Speed TP, Dudoit S. Normalization of RNA-seq data using factor analysis of control genes or samples. Nature biotechnology. 2014;32(9):896-902.

5. Love MI, Huber W, Anders S. Moderated estimation of fold change and dispersion for RNA-seq data with DESeq2. Genome Biol. 2014;15(12):550.

6. Krämer A, Green J, Pollard J, Jr., Tugendreich S. Causal analysis approaches in Ingenuity Pathway Analysis. Bioinformatics (Oxford, England). 2014;30(4):523-30.

7. Breuer K, Foroushani AK, Laird MR, Chen C, Sribnaia A, Lo R, et al. InnateDB: systems biology of innate immunity and beyond-recent updates and continuing curation. Nucleic Acids Res. 2013;41(D1):D1228-D33.

8. Langfelder P, Horvath S. WGCNA: an R package for weighted correlation network analysis. BMC Bioinformatics. 2008;9:559.

9. Bosco A, Ehteshami S, Stern DA, Martinez FD. Decreased activation of inflammatory networks during acute asthma exacerbations is associated with chronic airflow obstruction. Mucosal Immunol. 2010;3(4):399-409.

10. Leek JT, Johnson WE, Parker HS, Jaffe AE, Storey JD. The sva package for removing batch effects and other unwanted variation in high-throughput experiments. Bioinformatics (Oxford, England). 2012;28(6):882-3.

11. Liu BH, Yu H, Tu K, Li C, Li YX, Li YY. DCGL: an R package for identifying differentially coexpressed genes and links from gene expression microarray data. Bioinformatics (Oxford, England). 2010;26(20):2637-8.

12. Subrata LS, Bizzintino J, Mamessier E, Bosco A, McKenna KL, Wikstrom ME, et al. Interactions between innate antiviral and atopic immunoinflammatory pathways precipitate and sustain asthma exacerbations in children. J Immunol. 2009;183(4):2793-800.

13. Kauffmann A, Gentleman R, Huber W. arrayQualityMetrics--a bioconductor package for quality assessment of microarray data. Bioinformatics (Oxford, England). 2009;25(3):415-6.

14. Dai M, Wang P, Boyd AD, Kostov G, Athey B, Jones EG, et al. Evolving gene/transcript definitions significantly alter the interpretation of GeneChip data. Nucleic Acids Res. 2005;33(20):e175.

15. Lu J, Kerns RT, Peddada SD, Bushel PR. Principal component analysis-based filtering improves detection for Affymetrix gene expression arrays. Nucleic Acids Res. 2011;39(13):e86.

16. Ritchie ME, Phipson B, Wu D, Hu Y, Law CW, Shi W, et al. limma powers differential expression analyses for RNA-sequencing and microarray studies. Nucleic Acids Res. 2015;43(7):e47.
