# Supplementary figures and images for: LPS binding protein and activation signatures are upregulated during asthma exacerbations in children
Source: Respir Res. 2023 Jul 12;24:184. doi: 10.1186/s12931-023-02478-3 (PMC10337076; doi:10.1186/s12931-023-02478-3)

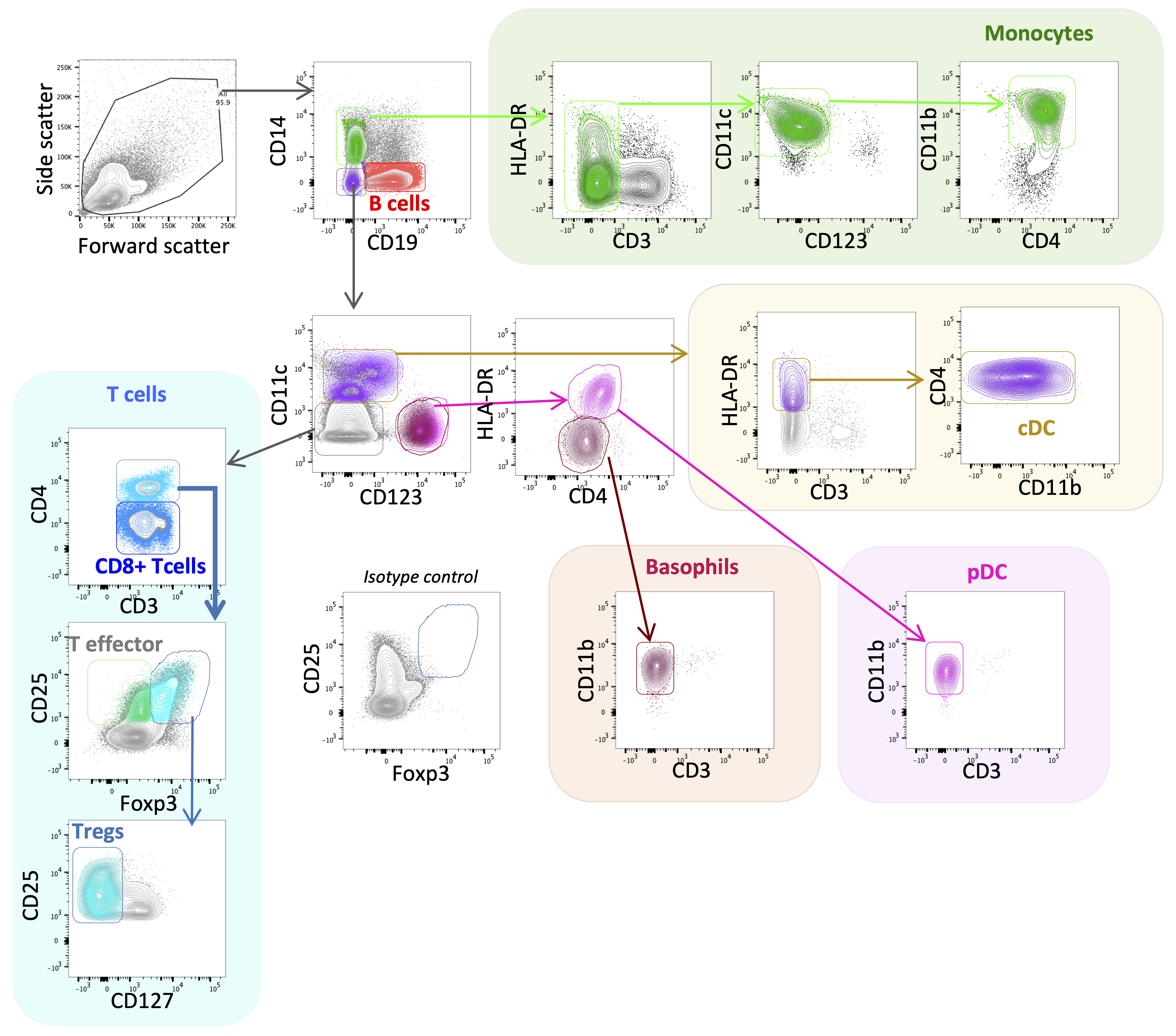

Supplement: Supplementary file 2 — Additional file 2: Fig. 1. Exemplary gating strategy for all subsets analysed. Example gating strategy from acute peripheral blood mononuclear cells labelled with a panel of antibodies to identify lymphoid and myeloid cells subsets [file 12931_2023_2478_MOESM2_ESM.tiff]

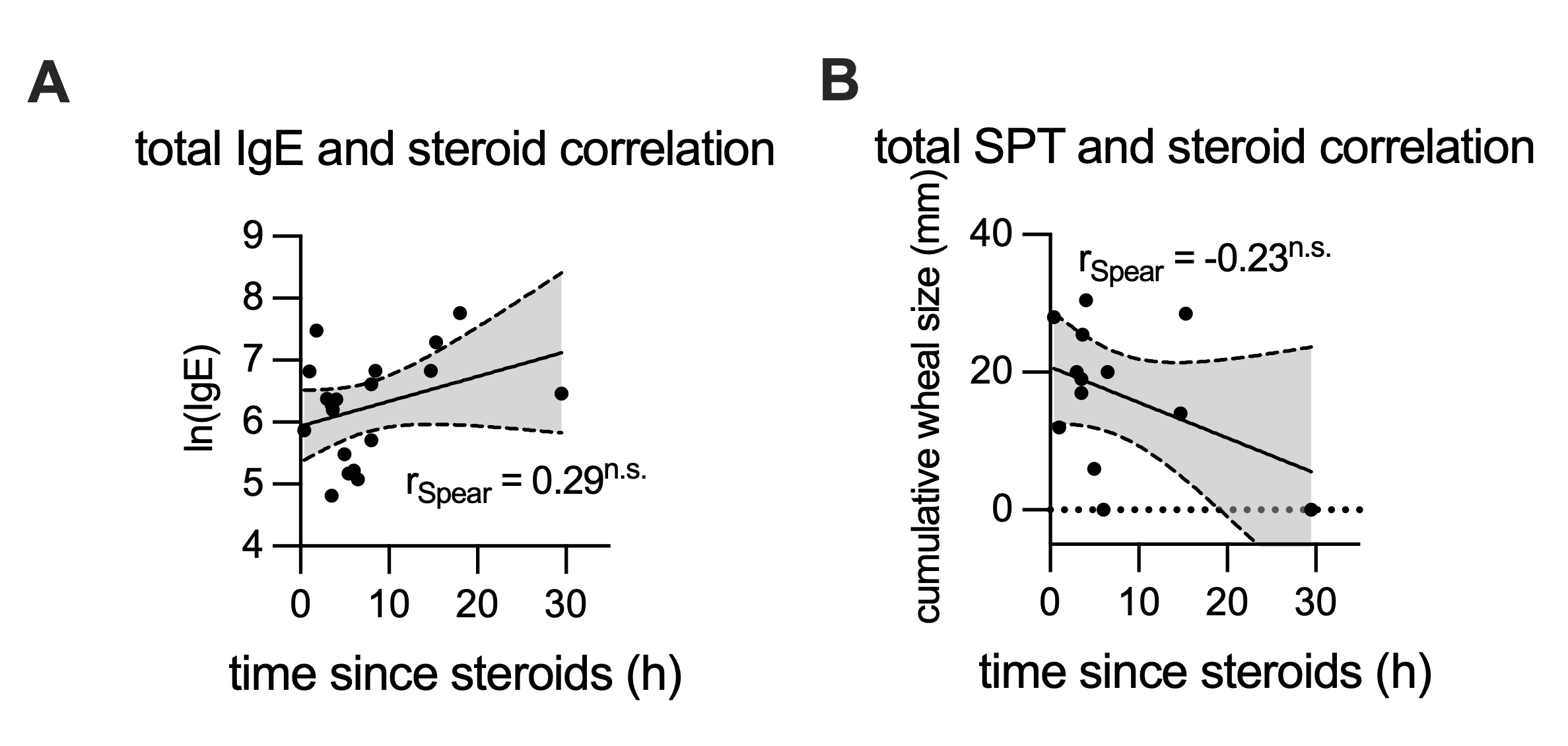

Supplement: Supplementary file 3 — Additional file 3: Fig. 2. Correlation with Atopy readouts and systemic glucocorticoid treatment. Total serum IgE and SPT cumulative wheal size did not corelate with time since administration of systemic glucocorticoids [file 12931_2023_2478_MOESM3_ESM.tiff]

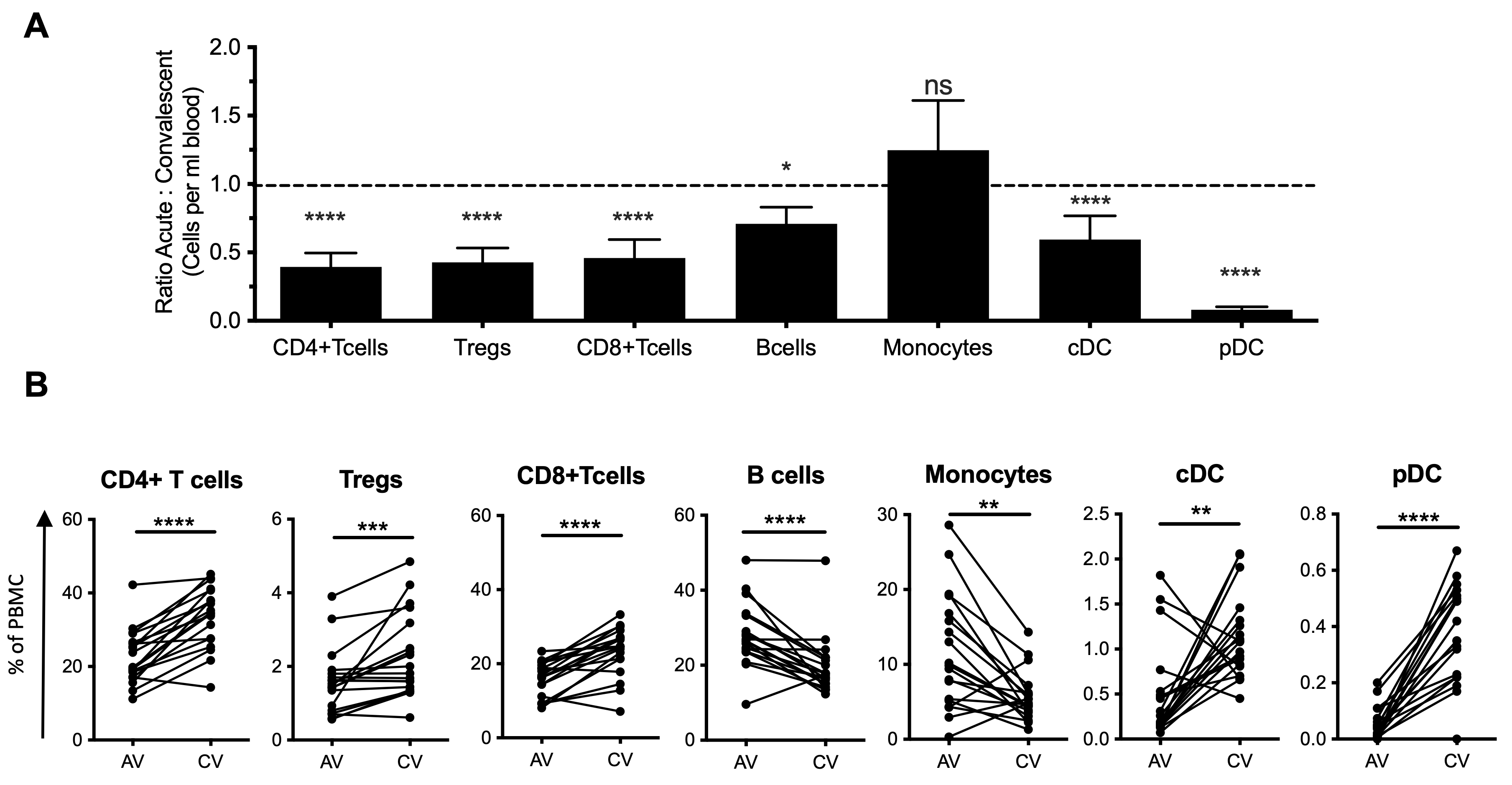

Supplement: Supplementary file 4 — Additional file 4: Fig. 3. Inflammatory cells are trafficking and leaving the peripheral blood. Peripheral blood mononuclear cells (PBMC) were sampled from atopic asthmatics at presentation to hospital Emergency during an exacerbation, the acute visit (AV), and following recovery at the convalescent visit (CV). Multi-colour flow cytometry was employed to quantify inflammatory cell subsets, in (A) ratio of acute: convalescent (cells per ml blood), data are represented as mean ± SEM, and (B) cellular frequency (percentage) of PBMC. The P-values are derived from a Wilcoxon test for paired analysis. ****,<0.0001, ***,<0.001, **,<0.01, *,<0.05 [file 12931_2023_2478_MOESM4_ESM.tiff]

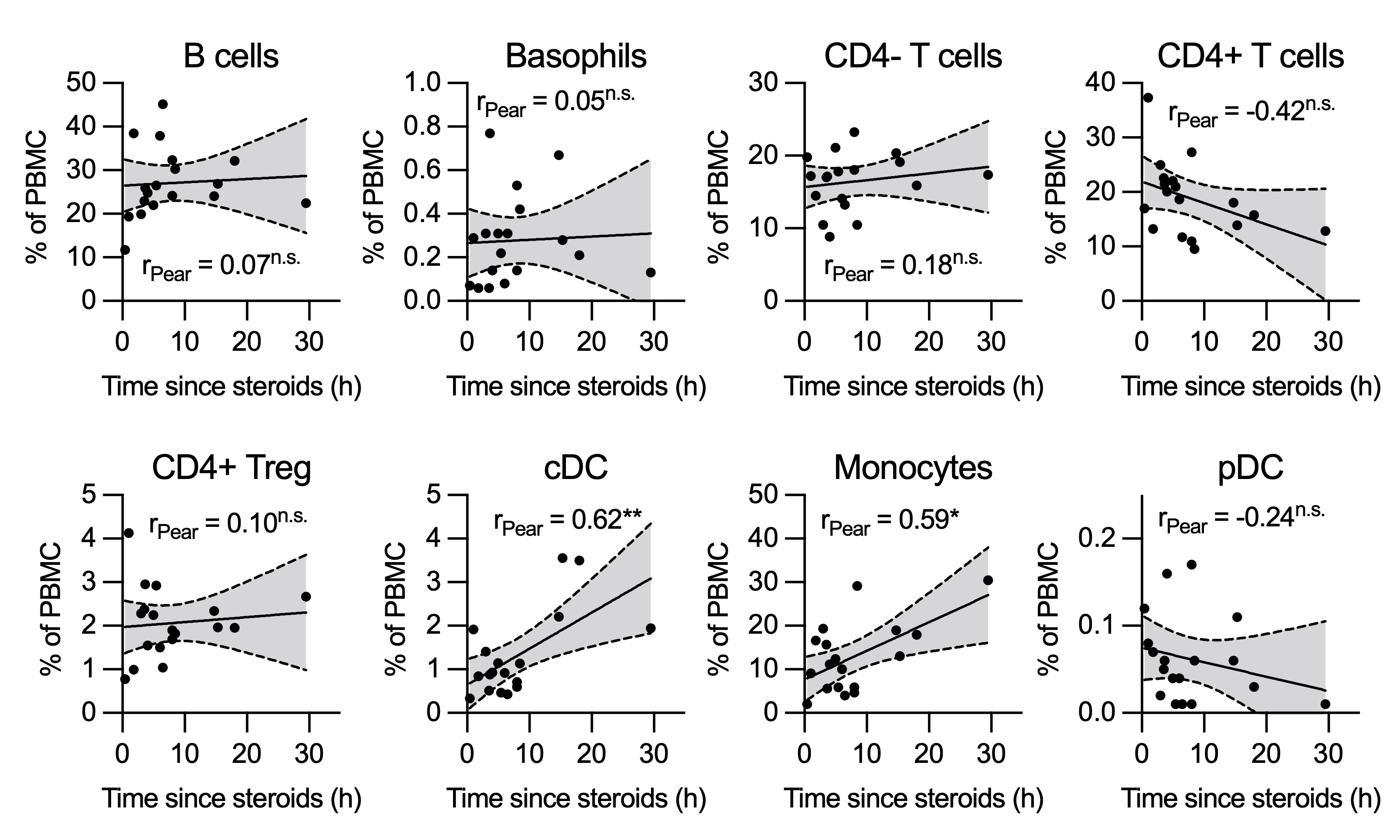

Supplement: Supplementary file 5 — Additional file 5: Fig. 4. Correlation with cell subset abundance during the acute event and systemic glucocorticoid treatment. Abundance of immune cell subsets correlated with time since administration of systemic glucocorticoids. Correlation was assessed using Pearson’s parametric correlation on samples for which data on steroid treatment was available, N = 18, **, p < 0.01, *, p < 0.05 [file 12931_2023_2478_MOESM5_ESM.tiff]

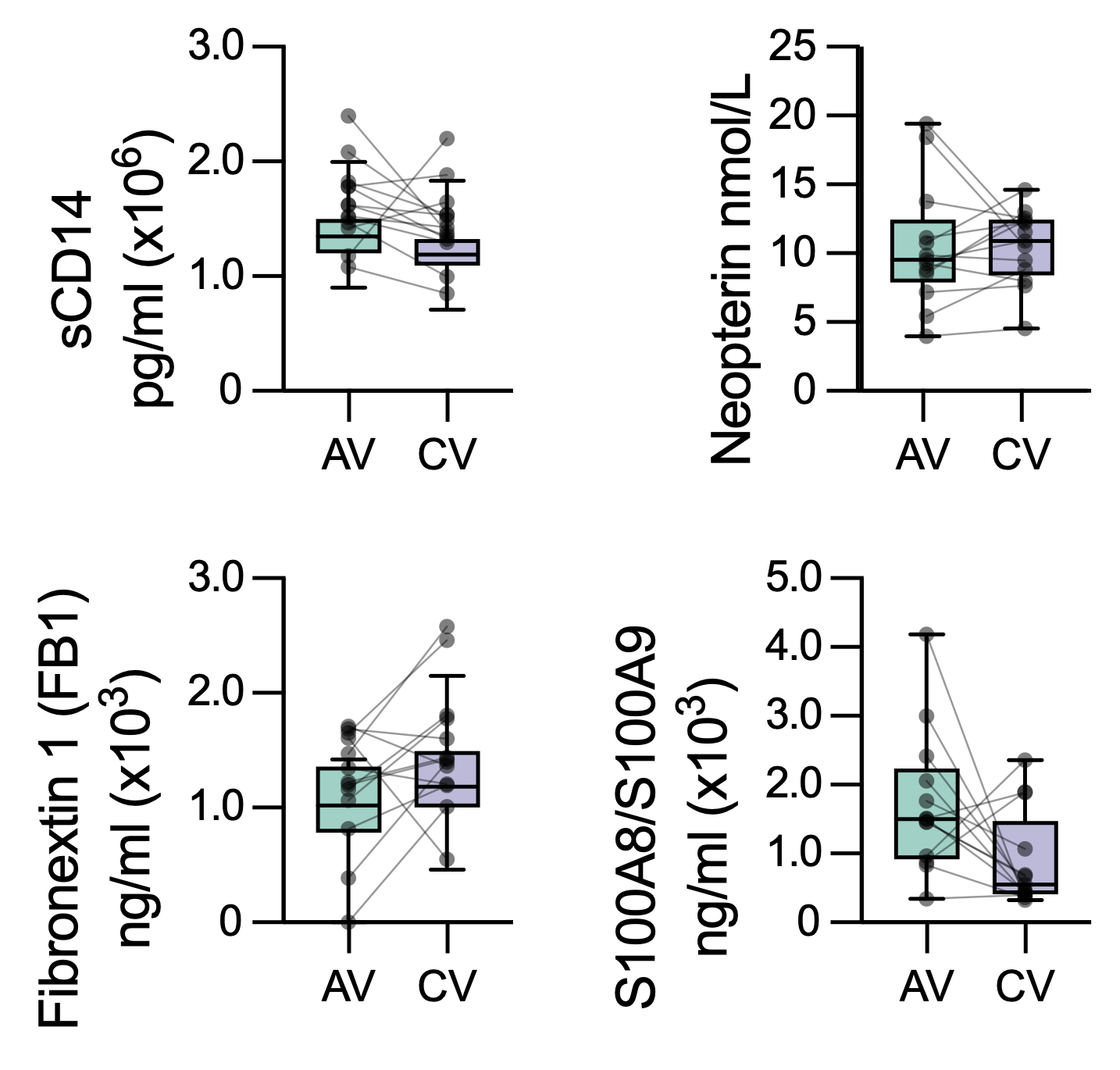

Supplement: Supplementary file 6 — Additional file 6: Fig. 5. Plasma levels measured of soluble CD14 (sCD14), neopterin, fibronectin 1 (FN1) and S100A8/S19 [file 12931_2023_2478_MOESM6_ESM.tiff]

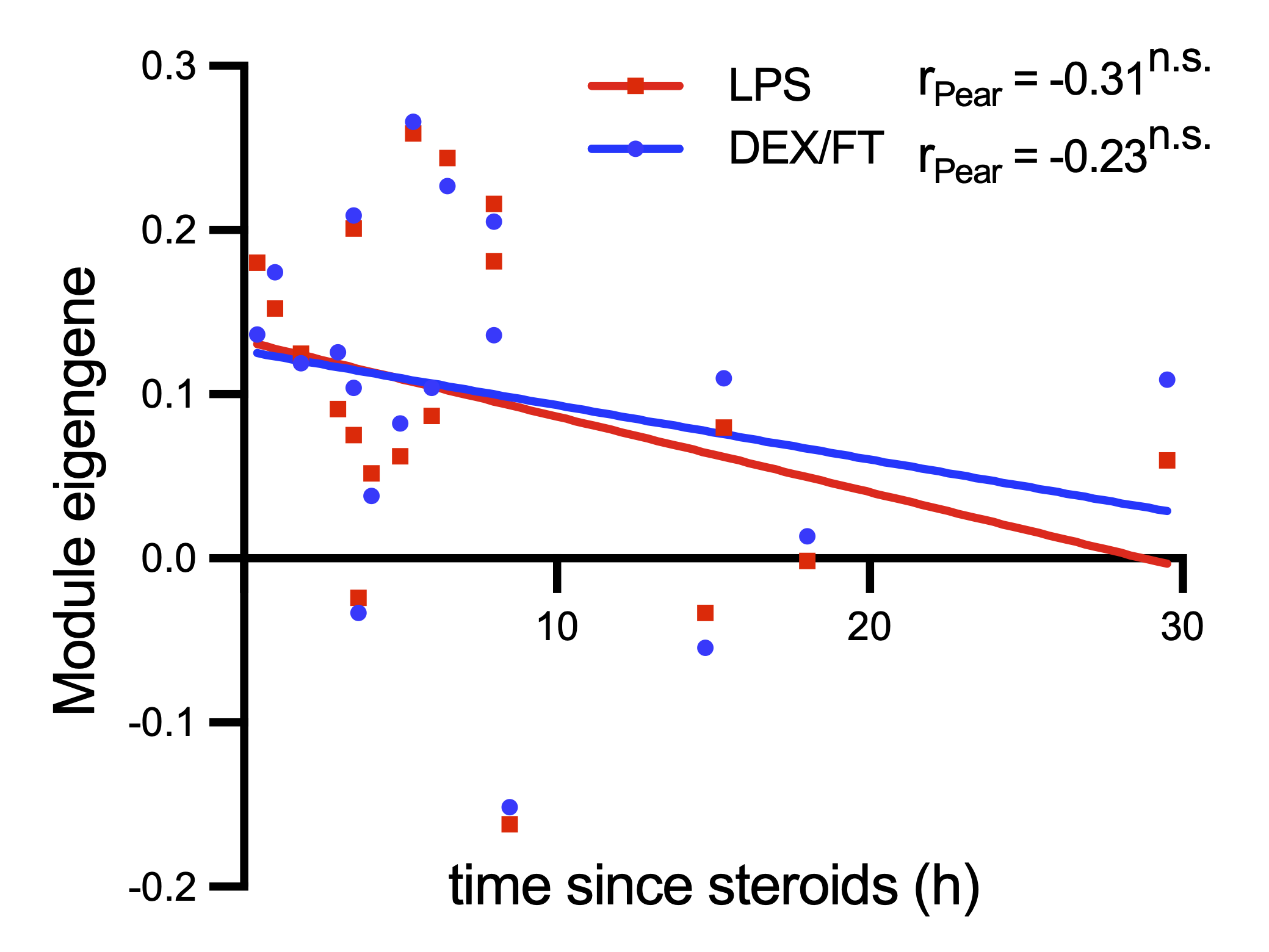

Supplement: Supplementary file 7 — Additional file 7: Fig. 6. Correlation of LPS and steroid module eigengene signatures from the upstream regulator analysis during the acute event and time since glucocorticoid treatment. Time since steroid administration does not correlate with either the DEX/FT or the LPS signature. Correlation was assessed using Pearson’s parametric correlation, n.s. = not significant [file 12931_2023_2478_MOESM7_ESM.tiff]

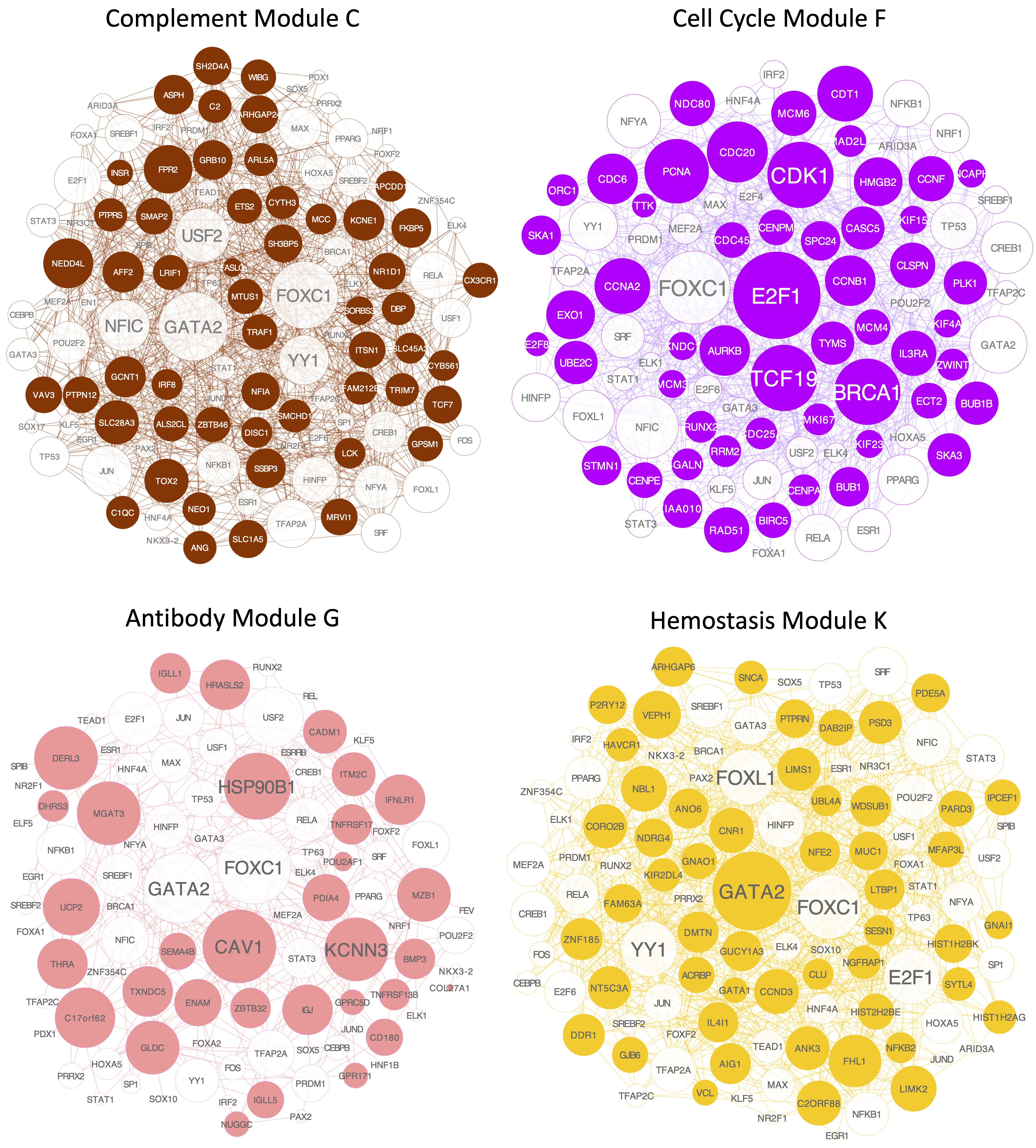

Supplement: Supplementary file 8 — Additional file 8: Fig. 7. Reconstruction of the wiring diagram of modules C, F, G and K. Coloured node = present in the original network (zero order network), white node = transcription factors [file 12931_2023_2478_MOESM8_ESM.tiff]

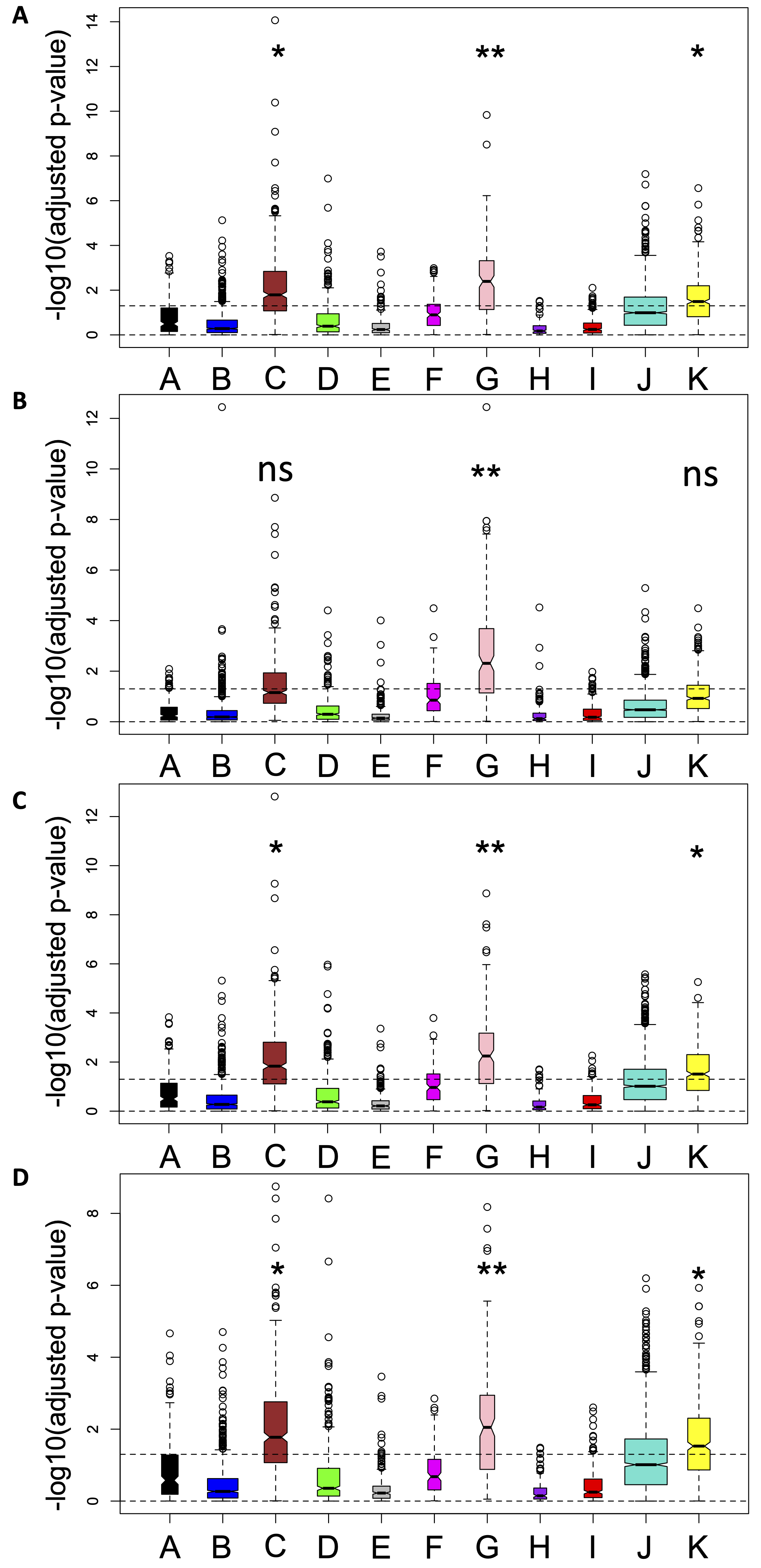

Supplement: Supplementary file 9 — Additional file 9: Fig. 8. Identification of exacerbation-associated modules with or without adjustment for cellular composition. The data was analysed with DESeq2/RUVSeq and adjusted for cellular composition, in (a) unadjusted networks, (b) adjustment for proportions of monocytes, (c) adjustment for proportions of B cells, and (d) adjustment for proportions of T cells. The dashed horizontal line indicates an adjusted p-value < 0.05. **median adjusted P-value < 0.01, *median adjusted P-value < 0.05, ns = not significant [file 12931_2023_2478_MOESM9_ESM.tiff]
